# A Nationwide Survey on Patient Empowerment in Pediatric Inflammatory Bowel Disease in Germany

**DOI:** 10.3390/children10121904

**Published:** 2023-12-09

**Authors:** Kalina Kaul, Stefan Schumann, Cornelia Sander, Jan Däbritz, Jan de Laffolie

**Affiliations:** 1 Department of General Pediatrics and Neonatology, University Children’s Hospital, University Giessen, 35392 Giessen, Germany; kalina.kaul@paediat.med.uni-giessen.de (K.K.); jan.delaffolie@paediat.med.uni-giessen.de (J.d.L.); 2 German Crohn’s Disease and Ulcerative Colitis Association (DCCV), National Association for Chronic Inflammatory Diseases of the Digestive Tract, 10179 Berlin, Germany; csander@dccv.de; 3 Greifswald University Medical Center, Department of Pediatrics, 17475 Greifswald, Germany; jan.daebritz@med.uni-greifswald.de

**Keywords:** pediatric inflammatory bowel disease, Crohn’s disease, ulcerative colitis, parents, chronically ill children, survey, patient empowerment

## Abstract

Background: The incidence/prevalence of pediatric inflammatory bowel disease (IBD) is increasing. IBD places a significant burden on young patients during important developmental stages and affects their families. Children and adolescents with IBD require increased support from health care services. However, little is known about the additional support needed and how to provide it. Therefore, a large survey was conducted with a focus on patient empowerment. Methods: For the anonymous survey, called CEDNA, a patient questionnaire for adolescents with IBD and a questionnaire for parents of children and adolescents with IBD were made available throughout Germany (distributed n = 2810). Questions covered various aspects of coping with the disease, utilization of care, use and need of information and communication services, and how information should be provided. Results: From October 2021 to April 2022, 1158 (n = 708 parents (61.1%), n = 450 patients (38.9%)) questionnaires were completed. The results show a deficit in pediatric IBD care and revealed a large gap in knowledge about core IBD topics (e.g., 74.8% of patients feel poorly informed about transition, 62.4% know little about patient organizations and self-help groups, and 54.7% have little information about preventive health measures), indicating a low level of health literacy among affected children and adolescents. Conclusion: Pediatric IBD poses significant challenges for patients, their families, and healthcare teams. By empowering patients and families, and providing targeted information and communication tailored to the age of the child or adolescent and the needs of the parents, care can be improved and better adapted to the needs of patients. Actions would include providing quality information online through scientific societies and patient organizations and facilitating self-management in adolescents.

## 1. Introduction

Inflammatory bowel disease (IBD), with its major forms Crohn’s disease (CD) and ulcerative colitis (UC), affects approximately 7 million people worldwide [1]. While incidence and prevalence figures for adult IBD appear to be stabilizing at high levels in Western industrialized countries, pediatric IBD (PIBD) continues to increase worldwide [2,3,4]. The most recent estimate for Germany, based on hospital claims data, is an incidence of 17.41 (95% confidence interval 15.08–20.10) per 100,000 insured children and adolescents per year in 2012, compared with 13.65 (CI 11.62–16.01) in 2009. The prevalence was estimated to be 66.29/100,000, which is higher than the prevalence found in most studies on this topic worldwide [5].

Pediatric inflammatory bowel disease (PIBD) is associated with a more invasive and complicated phenotype with more extensive inflammation, significant limitations in health-related quality of life (HRQoL), and a high risk of developmental impairment [6]. This requires more intensive and multimodal modern immunomodulatory and immunosuppressive therapy involving a multidisciplinary team, patients, families, and peers [6,7].

The already challenging tasks of growth and development during the crucial transition to adulthood can be severely disrupted by disease symptoms or therapy-related difficulties, leading to limitations in school and daily activities, as well as psychosocial comorbidities [8,9,10,11]. As a result, affected individuals have increased utilization of health care services [8,12].

Acceptance and positive coping strategies are promoted by patient, family, and environmental resilience factors [13]. Successful coping with chronic illness has long-term consequences that extend well into adulthood [14]. The critical issues that children and adolescents face as a result of their illness are also important for the healthcare teams involved: Achieving shared therapeutic goals, successful autonomy and self-management, good patient adherence with peer support [15,16], and effective communication and relationships with the care team [17]. This significantly reduces the risk of either undertreatment or overtreatment, which can lead to persistent disease or complications.

Little is known about the additional support needs of patients and their families and how these can be met. The aim of the CEDNA survey was to reflect on the current care situation of children and adolescents with IBD in Germany and to create a database for the improvement process of communication between healthcare teams patients and other stakeholders in regional and international IBD healthcare systems. These are elements that contribute to patient empowerment. The needs assessment will be used to identify ways to improve existing medical structures and to develop patient empowerment services. The results of the survey and the empowerment concepts developed serve to improve the patient competence of each IBD patient and his or her social environment. Overall, this paper is about patient empowerment in children and adolescents with IBD.

Based on exploratory data analysis of the CEDNA survey, we aimed to gain insights from the patient- and disease-specific contexts regarding treatment and disease management to improve collaboration between healthcare teams affected families, and patient associations.

## 2. Methods

For the anonymous CEDNA survey (an acronym for IBD needs analysis), two versions of the questionnaires were distributed from October 2021 to April 2022: one for adolescent patients with IBD between 12 and 17 years of age (28 questions) and one for parents of children and adolescents with IBD up to and including 17 years of age (41 questions). The survey asked adolescent patients with IBD and parents of adolescent patients with IBD about their wishes and needs for additional support. The focus was on socio-demographic and disease-specific factors and their influence on treatment-related issues. These included coping mechanisms, information status and deficits, utilization and need for treatment and counseling services, and age- and disease-appropriate communication. In the parent questionnaire, no distinction was made as to whether the father or the mother completed the questionnaire. The questionnaires were provided in both printed and online versions (via the online survey tool LimeSurvey, 2021–2022) together with a patient information leaflet.

The questions covered aspects of medical and psychological care in IBD, coping, the need for information and counseling services, and how information should be provided. All topics were asked of both patients and parents, as there was no requirement that both matched groups complete the questionnaire. 

The CEDNA survey was part of the Patient Empowerment sub-project of the overall German project to improve the quality of care for pediatric IBD patients, CED-KQN (Big Data eHealth—Improving the Care of Children and Adolescents with IBD, www.cedkqn.de, accessed on 31 October 2023). The CED-KQN project was funded by the Joint Federal Innovation Committee G-BA (www.g-ba.de, accessed on 31 October 2023) under grant number 01VSF17054 and was recognized as a lighthouse project at the German Congress for Health Services Research in Berlin in 2023. This has already resulted in a publication dealing with the availability of pediatric IBD care services in Germany and the accessibility of centers [18].

The CED-KQN project explored different aspects of health services research in children and adolescents with IBD in the context of big data and e-health: A controlled, cluster-randomized, two-arm study (CLARA study) was conducted on the CEDATA GPGE patient registry, one of the largest patient registries in the world with important information on disease presentation, treatment and progression, to investigate whether registry-based feedback can improve the care of children and adolescents with IBD. Based on this, new technologies and innovative approaches should be used to improve the care of children and adolescents with IBD and to fill existing gaps in care. New information technology approaches for registry-based clinical decision support algorithms were developed and the underlying patient registry information technology was adapted to ensure a successful transition to adult medicine. In the Patient Empowerment sub-project, in addition to the CEDNA survey, a patient empowerment app (CEDMO App) was developed to help children and adolescents document their disease in everyday life. The CEDMO App is a digital medium designed to appeal to patients over the age of 12, who can use the app to quickly record key information about their condition, diet, weight, or doctor’s appointments. They can enter school absences and personal notes and upload pictures. The CEDMO App can be linked to the CEDATA GPGE registry and patient data can be transferred to it with consent. This supports the patient empowerment approach by the treating physician and was also asked in the CEDNA survey about preferred media for patients.

Experts from the Westbrandenburg Hospital, the German Crohn’s Disease and Ulcerative Colitis Association (DCCV), the Society for Pediatric Gastroenterology and Nutrition (GPGE), the IBD patient registry CEDATA- GPGE network of pediatric IBD clinics, the University Medical Center Rostock and the University Children’s Hospital Giessen were involved in the development of the questionnaires. A working group consisting of specialized pediatric gastroenterologists, scientific staff of the CED-KQN project, staff of the DCCV, and the study center of the University Children’s Hospital Giessen was formed. For more than a year (November 2018–February 2020), the working group collected IBD-specific topic areas for a questionnaire, based on both professional experience and previous patient surveys of the participating experts. The topics of the questionnaire were continuously filled with specific questions about IBD and patient empowerment.

The developed questionnaires were tested by a multidisciplinary team (with different levels of experience in the field of PIBD) and patients as well as their relatives during consultation hours at the Westbrandenburg Hospital. In addition, the questionnaires were modified based on further feedback from additional patients during clinic hours and tested again by the medical PIBD teams in Rostock and Giessen. Templates for the inclusion of patient empowerment in the spectrum of PIBD were not available in Germany and only to a limited extent internationally. Therefore, the use of existing questionnaires was not an alternative. Since a formal validation was not possible, an exploratory hypothesis-generating analysis was performed.

In addition to the initial development, comprehensibility was tested in interviews with patients and multidisciplinary medical teams. The patient questionnaire contained fewer questions than the parent questionnaire. It was written in a language appropriate for adolescents, and the response options were less extensive. Emerging differences between the patient and parent questionnaires were intended to discover differences in the self-perception of IBD patients and the perception of their peers. In addition, the parent questionnaire provided an opportunity for the families of IBD patients to describe their burden, the counseling services they use, and the challenges of dealing with the disease in the family.

Questions covered aspects of the disease (e.g., comorbidities), medical and psychological care (e.g., mental or physical problems), coping with the disease (e.g., activities or participation in a support group), and ways of learning about the disease (e.g., sources of information, use of treatment and counseling services).

The basic idea of the needs analysis was to get an overview of the needs and wishes of IBD patients and their families and the challenges they face in their daily lives. Socio-demographic data were collected to characterize the anonymous data and to derive age-specific offers from the results. Patient- and disease-specific factors (including medical care, disease duration, and disease activity) were added by the working group to identify relevant correlations.

Inclusion criteria were patients and parents of patients with diagnosed IBD (CD, UC, IBD unclassified) in children and adolescents up to and including 17 years of age. There were no patient or parent exclusion criteria nor were there any dropout criteria or exclusion of a patient or parent during the CEDNA survey.

Printed questionnaires were distributed throughout Germany via the patient organization DCCV (410 copies to adult members with consent to be contacted and their children with IBD up to 17 years of age) and via the CEDATA-GPGE patient register (323 copies to the address database of patients and their parents with consent to be contacted and who were not older than 17 years of age). The GPGE network clinics distributed 2077 printed questionnaires directly to patients or their parents. A total of 80 GPGE network clinics throughout Germany (see www.gpge.eu, accessed on 31 October 2023) were contacted and asked to support the CEDNA survey. Due to the anonymity of the survey, it was not possible to determine how many centers participated. The online distribution was done through these media: CEDATA-GPGE newsletter to pediatric gastroenterologists in clinics and practices, posts on the DCCV Facebook page, announcement on the DCCV website, in the DCCV newsletter to members of the patient association, a microsite for the CEDNA survey on www.cedmo.de (CEDMO app for children and adolescents with IBD, accessed on 31 October 2023), study call page on www.abbvie-care.de (accessed on 31 October 2023) and a study called in the “leben-mit-ced.de” forum of AbbVie (no funding was provided) and announcement on the project website www.cedkqn.de (accessed on 31 October 2023).

There were no reliable epidemiologic data on children and adolescents with IBD in Germany to calculate the number of cases. Therefore, a response rate of 300 questionnaires was estimated. This number was derived from the number of patients who could be reached by mail through the DCCV (about 400) and the CEDATA GPGE registry (about 300), plus the distribution through the 80 GPGE centers (with an estimated 15 IBD patients per center, total 1200) and the online survey call (estimated 1100). The estimated response rate was 10%.

There was no compensation or other incentive to participate in the survey.

The printed questionnaires were digitized into Excel (Office 365, version 2022), and the responses to the online questionnaires were imported from LimeSurvey into Excel. Both Excel lists were analyzed using SAS statistical software (version 9.4) for descriptive analysis.

No personal information about the participants was available from the survey. All participating institutions that distributed the survey, as well as the patients themselves, were continuously advised not to include any unique names on the questionnaires, which were adhered to.

## 3. Results 

### 3.1. Response and Characteristics of Participants

A total of 1158 participants completed the CEDNA survey: 450 patient questionnaires (310 printed, 140 online; patients n = 450) and 708 parent questionnaires (454 printed, 254 online; parents n = 708) were returned. With 764 printed questionnaires returned, the response rate was 27%. The online response rate could not be determined because there was no tracking of site visitors or site of origin. The survey results regarding gender, region, age, and disease spectrum reflect the estimated epidemiology and disease spectrum of PIBD in Germany, indicating that the CEDNA is a representative survey. In addition, the responses regarding factors such as comorbidities and duration of diagnosis are similar to the overall population of children and adolescents with IBD. Considering the prevalence of the disease, this is the largest survey conducted on this population worldwide.

In the presentation of the results, the term “patients” refers to the surveyed target group between the ages of 12 and 17. The term “parents” refers to all parents who completed the questionnaire for their sick children up to the age of 17. Data from missing or incorrect responses were not included. The magnitude of these incorrect or missing answers for the patient questionnaires was median = 0.15, P25 = 0.089 to P75 = 0.19. For the parent questionnaires, the values were median = 0.22, P25 = 0.08 to P75 = 0.27.

The characteristics of the patients who participated in the CEDNA survey themselves and of the patients whose parents participated in the CEDNA survey are shown in Table 1.

### 3.2. Care Structure and General Satisfaction with Care

Most parents (98.9%, n = 611) confirmed the existing care by a pediatric gastroenterologist and only 1.1% said “no” to the existing care by a pediatric gastroenterologist.

In the 12 months prior to the survey, the primary contact for all questions about the disease was a pediatric gastroenterologist (patients 90.6%, n = 406; parents 96.7%, n = 210). The general pediatrician was the primary contact for 14.0% (n = 406) of the patients (1.4% parents, n = 210). Other contacts were the family doctor (patients 4.4%, n = 406; parents 0.5%, n = 210) and an adult gastroenterologist (patients 3.0%, n = 406; parents 1.0%, n = 210). According to the parents, none of the patients were left without a main contact, but 1.2% (n = 406) of the patients reported that they had no main contact.

The parents (n = 612) reported that the travel time to the specialist and/or specialized clinic for IBD was “30–60 min” (50.5%), “less than 30 min” (29.6%) and “more than 60 min” (19.9%). In their home area (up to 100 km away), 55.8% of parents knew of a specialist and/or a specialized clinic for IBD patients. One-third of the parents (37.6%, n = 607) knew two to five specialists, and 1.5% “more than 5”. 5.1% of parents did not know any IBD specialists. 

Most parents (n = 611) were satisfied with the medical care of their child with IBD: 50.6% were “completely” satisfied and 44.8% were “mostly” satisfied. Only “a little” satisfied was 4.1% and “not at all” satisfied was 0.5%. In addition, most patients (93.7%, n = 399) were satisfied with their medical care and only 6.3% (n = 399) reported not being satisfied.

Parents of affected children (n = 558) were asked if they had ever been denied coverage by the insuring health plan or subsidy. This occurred in 8.8% of cases, with 91.2% of respondents reporting that full coverage was provided. In general, 84.0% of respondents (n = 587) were satisfied with their health plan, while 16.0% were dissatisfied.

### 3.3. Medical Societies and Patient Associations 

More than half of the parents (n = 537, 55.0%) did not know whether their child’s medical data had already been recorded in the central IBD patient registry CEDATA-GPGE of the German-speaking Society for Pediatric Gastroenterology and Nutrition (GPGE, www.gpge.eu, accessed on 31 October 2023). 28.2% of respondents said that they remembered giving their consent for data to be collected, and 16.8% knew that their data would not be collected in the registry. In addition to the GPGE, awareness of other professional and patient associations was assessed (multiple answers were possible): 84.0% of the parents (n = 537) knew about the German Crohn’s and Ulcerative Colitis Association (DCCV, www.dccv.de, accessed on 31 October 2023), 30.0% about the German Nutrition Society (DGE, www.dge.de, accessed on 31 October 2023) and 16.8% about the GPGE. Less well known were the “Competence Network Inflammatory Bowel Disease” (www.kompetenznetz-darmerkrankungen.de, accessed on 31 October 2023) (3.9%), the “German Self-Help Association for Ostomates and their relatives” (www.ilco.de, accessed on 31 October 2023) (3.2%), the “Transition Program” (e.g., the Berlin Transition Program, www.btp-ev.de, accessed on 31 October 2023) (3.0%) and the “Children’s Network” (www.kindernetzwerk.de, accessed on 31 October 2023) (3.0%).

### 3.4. Physical Problems and Psychological Support

When physical problems related to IBD occurred, 81.7% (n = 409) of patients and 89.3% of parents (n = 607) reported that their children or they themselves “always” turned to someone for support. 11.5% of patients (n = 409) and 7.1% of parents (n = 607) reported that they were able to reach someone “sometimes”. The statement “rather not available” was made by 6.8% (n = 409) of the adolescents and 3.6% of the parents (n = 607). 

To the question “who could be contacted in case of physical problems”, “doctor” was chosen by 71.8% (n = 400) and “parents and family” by 93.8% (n = 400) of the patients, among the parents (n = 609) by 70.3% and 95.7% (n = 609), respectively. “Friends” was selected by 27.8% (n = 399) (parents 18.1%, n = 609).

In addition to physical problems, mental or emotional problems related to IBD were also addressed. 79.3% (n = 401) of patients reported that someone was “always” available (parents 90.3%, n = 589). “Now and then” was answered by 14.0% (n = 401) of patients (parents 7.5%, n = 589), and “never” by 6.7% (n = 401) (parents 2.2%, n = 589).

When asked who was available, 88.2% (n = 391) of patients answered, “parents and family” (parents 96.7%, n = 600), 36.3% (n = 391) answered “doctor” (parents 43.2%, n = 600), and 45.0% (n = 391) answered “friends” (parents 25.8%, n = 598).

### 3.5. Coping with the Disease

In the three months prior to the survey, 59.2% (n = 402) of patients and 52.7% (n = 603) of parents were able to cope with IBD-related emotions “most of the time”, 33.1% (parents 38.3%) “completely”, 6.2% (parents 7.3%) “only a little” and 1.5% (parents 1.7%) “not at all”. Parents were asked how strongly they experienced the listed emotions related to their child’s IBD at the current stage of the disease, and adolescents were asked how they felt when thinking about their IBD: 31.8% (parents 73.3%, n = 592) felt uncertain and 74.0% (parents 75.3%, n = 575) felt confident. Regarding the future of their child, 66.6% (n = 593) of the parents were afraid of what would happen.

Anxiety per se was reported by 17.4% (n = 450) of patients (parents 66.6%, n = 593), exhaustion by 33.9% (n = 450) (parents 53.4%, n = 581). The patients also stated: Nervousness by 26.9% (n = 450) (parents 48.6%, n = 584), helplessness by 7.4% (n = 450) (parents 47.7%, n = 576), and being overwhelmed by the situation by 18.6% (n = 450) (parents 43.8%, n = 576). 7.2% of patients (n = 450) were disheartened (parents 40.4%, n = 577). Feelings of abandonment were reported by 6.1% (n = 450) of patients (parents 25.0%, n = 577) and feelings of guilt by 28.8% of parents (n = 577). Patients expressed a feeling of calmness in 68.6% (n = 450) (parents 40.4%, n = 565). Great worry was reported by 45.0% of patients (n = 586), depression by 18.6% (n = 450) (parents 57.2%, n = 577), and shame by 14.4% (n = 450) (parents 5.7%, n = 579). Patients affirmed “I feel lonely” in 13.8% of responses (n = 450), “I feel sad” in 28.3% (n = 450), “I feel insecure” in 37.3% (n = 450) and “I think everything will be fine” 86.0% (n = 450).

### 3.6. Dealing with Inflammatory Bowel Disease

Patients were asked if they used certain options to cope with IBD, and parents of patients were asked how often their children used these options. The most common way for patients to cope with the disease was to enjoy hobbies, and the least common activity was to participate in a support group. All responses regarding coping are shown in Table 2.

### 3.7. Counseling and Information Services on IBD

More than half of the patients (51.8%, n = 388) and half of the parents (64.1%, n = 588) felt sufficiently informed about IBD, with “most” and 37.6% (n = 388) of patients and 26.4% of parents feeling “completely” informed. In contrast, only 8.8% (n = 388) felt “not very” informed (parents 8.3%), and 1.8% (n = 388) felt “not at all” informed (parents 1.2%). 

Patients and parents were asked which IBD-related topics they felt knowledgeable about, which topics they would like to learn more about, and which treatment and counseling services they have used in the past. They were also asked who should provide information about IBD and how they would like to receive this information. The responses to these questions are shown in Table 3.

Patients’ and parents’ perceptions of the trustworthiness of different sources of information about IBD are summarized in Table 4.

Patients indicated whether a source was trustworthy and whether they had received information from that source. The most trusted source of information for patients was doctors, followed by professional journals and medical societies. Parents also indicated the trustworthiness of sources, ranging from very trustworthy to not trustworthy at all. Their preferred source of information was also doctors followed by patient associations and medical societies.

As the disease progresses, the patient’s needs and wishes change. Table 5 shows the different topics that were most likely to be of interest to parents at different stages of the disease. At diagnosis, over half of the parents wanted general information about IBD and its causes. In the first year after diagnosis, information on how to deal with school issues was most important, and later in the course of IBD, transition to adult medicine was the most important topic.

## 4. Discussion

The CEDNA survey is a comprehensive reflection of the care situation and needs of adolescents with IBD and parents of affected children and adolescents with IBD in Germany in 2022. The findings are grouped around key IBD issues that have direct implications for improving the quality of care, empowering patients, and families, aligning the activities of healthcare organizations, and counseling and information services. The results provide a starting point for optimizing care, improving the needs of patients with IBD and their families, and strengthening patient empowerment.

A pediatric gastroenterologist was available to 96.7% of parents (90.6% of patients). At first glance, this seems to reflect an adequate care situation.

However, given the time and distance that parents must travel to receive this care, it is associated with high costs and effort for patients and families, including time away from school or other activities. Overall, 50.5% of parents travel up to an hour or more to see an IBD specialist team. Thus, CEDNA confirms the finding of an outpatient and inpatient care deficit in pediatric and adolescent gastroenterology as presented by Zernickel et al. [19]. The hidden and invisible costs that the existing care structure imposes on patients and their families are growing. These are the reduced productivity of the patient and the family, as well as the lost time with the family due to the effort to be able to perceive the appropriate care [20]. This aspect is also reflected in the high percentage of patients’ parents who report fear of losing their jobs [21] or fear for their marriage and family.

The empowerment of patients and their families to better manage their disease—so-called patient empowerment—is repeatedly called for in review articles and studies. For example, it has been implemented in the US ImproveCare Now Learning Health System [22]. For other chronic diseases, such as diabetes mellitus, self-management, and patient empowerment are important to improve patient care. [23]. Behavioral interventions to empower patients can improve the way they manage their own chronic disease. Among other things, this can lead to more reliable medication adherence [24]. In a systematic review, Riemann et al. showed that “health literacy is associated with self-efficacy, health-related quality of life, and health care utilization in pediatric patients” [25]. One result of our survey is the lack of interest in self-help groups for PIBD. A scientific analysis of the reasons for this is still lacking. Further studies should try to find out the reasons to optimize the services and to develop future services.

The CEDNA survey showed a low level of interest from both patients (17.8%) and parents (10.7%) in learning more about patient organizations and support groups. This is a major barrier to patient empowerment in IBD. Only one in four patients and less than one in five parents feel well informed. When asked what topics patients and parents would like to learn more about, patient organizations and support groups ranked last in this survey. Previous studies on patient support groups [26] showed that 69% of affected families were aware of a support group, but only 16% used it. This severely limits their role in patient empowerment [27]. Parents of IBD patients, together with the multidisciplinary treatment team and peers, need to take on the role of facilitators of self-help so that support services should always be tailored to children, adolescents, and parents [28]. In contrast to the low interest of patients, parents, other patients, patient associations and support groups are the most trusted contacts for children and adolescents with IBD in the present study. One reason for this may be insufficient awareness of the role and usefulness of patient organizations, which needs to be addressed in future efforts. Another reason may be that patients and their support systems are under great stress from the indirect costs and efforts of organizing visits and medical issues, and do not feel they have the capacity or qualifications to engage in patient/self-help organization. A stressor for affected families may also be the lack of specialized medical care in rural areas with long distances and time to travel, as shown in our data.

Young patients have the greatest information gap regarding the causes of their IBD, as do the parents of patients. Therefore, there is a great need to provide easy-to-understand, age-appropriate, and comprehensive information and counseling specifically for this target group, including parents, peers, and relatives.

One focus of the survey was to determine the preferred way to receive information about the disease. The results show that digital communication of information about the disease is preferred by adolescent patients with IBD. This is in line with recent studies attributing growing importance to so-called online empowerment in our society [29,30] and explains why face-to-face self-help is much less popular according to the data of this survey. Patients are most interested in the Internet, YouTube, and educational films as information channels. A study of adolescents found that Internet use averages four hours per day and YouTube is the primary channel for watching movies [31]. Thus, the use of these platforms is extremely beneficial in disseminating disease knowledge to adolescents. In IBD, it is particularly important to provide low-threshold access to such sources and to provide information in a trustworthy and high-quality manner. This is an essential area for collaboration between the scientific community and patient advocacy organizations.

An Important issue for long-term low-risk disease progression and reduction of complications is the process of structured and supported transition from pediatric gastroenterology to adult gastroenterology. Previous studies have shown that a good transition has a positive impact on health-related quality of life [32,33]. However, deficits in knowledge about medications, self-management, and disease stages have previously been shown in adolescent patients with IBD [24,34]. To achieve the positive effects of a successful transition, this transition should be understood as a long-term, individualized process that begins in early adolescence [35]. In the present survey, only 0.9% of all parents reported that their child used a transition program. Only 25.2% of patients and 9.1% of parents feel well informed about these programs. 58.5% of patients and 22.4% of parents indicate a need for more information about transition programs, and 84.2% of parents express an increasing interest in the topic of transition as therapy progresses. These results demonstrate the current critical need for counseling services.

Regarding the psychosocial aspects of IBD, key questions were identified regarding feelings, processing, coping, and important influencing and resilience factors. The prevalence of mental disorders in children with IBD in the CEDNA study is low, up to 9.9%, compared to 15% of mental disorders in the KIGGS study [36]. However, the CEDNA study only collected data on diagnosed mental disorders, not on mental abnormalities. Other studies have also found an association between PIBD, depressive disorders, and quality of life [37,38]. Patients with PIBD have a 50% increased risk of developing depression [39]. A systematic review of psychosocial factors of resilience in young people with IBD [10] showed that resilience is largely unexplored in this context. In the CEDNA survey, young people largely reported being able to manage their emotions in relation to their IBD with positive externalizing behaviors in relation to coping style and resilience factors.

A limitation of the CEDNA survey is the lack of comparable preliminary studies on this pilot survey in the German and international literature. Therefore, iterative evaluations between patient groups including parents and the multidisciplinary team were conducted instead of a classical validation procedure. Due to the central focus of the survey on patient empowerment, and to facilitate responses and adapt to age-appropriate language, well-known questionnaires such as those on HRQoL in IBD (Impact III) were not used [40,41]. The choice of survey topics resulted from a compromise between the length of the questionnaires (to optimize response behavior) and the range of questions relevant to the patients involved.

The anonymous survey allows cross-sectional analysis at one point in time, and for the same reason, it was not possible to triangulate important parameters such as disease activity from a physician’s source. Conducting the CEDNA survey during the second half of the COVID-19 pandemic is also a limitation. Personal contacts were limited due to the need to protect the patient population with underlying immunologic disease and largely on immunomodulatory or immunosuppressive therapy from COVID-19 infection. In addition, the COVID-19 pandemic undoubtedly had a significant impact on the general lifestyle of the adolescents and parents surveyed. No additional questions specific to the COVID pandemic were asked. The results reflect the situation before or during the pandemic, and an analysis of this influencing factor was not performed separately. A representative distribution has been shown here, but the transferability to other countries is certainly limited, also due to other care systems and healthcare actors.

Despite these limitations, the recruitment estimate (300 questionnaires) was exceeded due to the strong commitment of patients and parents, with a response rate of 1158 questionnaires. A total of 2810 printed questionnaires were distributed and the survey was available online on five online platforms (DCCV, GPGE, CEDMO, CED-KQN, and AbbVie). Possible reasons for the difference between the number of questionnaires distributed and the actual number of completed questionnaires could be that patients and families who were not interested in participating were also contacted during the postal distribution, as no pre-selection was made based on anonymity. In addition, questionnaires were distributed directly to patients and parents at the clinical centers but were taken home to be completed and then, for various reasons, not completed or returned by mail. In addition, selection bias is possible because, although 80 clinical centers throughout Germany were asked to distribute the questionnaires, participation was voluntary and due to anonymity, it is not possible to determine which centers distributed the questionnaires to which patients and parents.

Our findings from the CEDNA survey are a first step in highlighting and describing how they deal with the emotions they experience in relation to their IBD. This can be used in future counseling services to achieve positive effects on health-related quality of life and increased resilience.

## 5. Conclusions

PIBD presents significant challenges for patients, their families, and healthcare teams. Peers and family members play an important role in addressing important patient needs. By involving patients and families (patient empowerment) through targeted information and communication, care can be more effectively tailored to the needs of patients and their families. Measures to improve care based on the results of CEDNA would include the provision of high-quality online information through scientific societies and patient organizations, and the facilitation of self-management in adolescents. So far, self-help groups and organizations are underutilized by families of children with IBD in Germany and need to be more directly integrated as a tool to improve patient self-management. The available evidence on information channels and communication tools, contact with treatment teams, and patient organizations will lead to more integrated concepts in patient empowerment and improvement of care for children and adolescents with IBD. The process of quality improvement needs to be monitored with subsequent studies.

## Figures and Tables

**Table 1:** The characteristics of patients who participated in the CEDNA survey themselves and of the patients whose parents participated in the CEDNA survey.

Responses in %					
	Patients	Parents		Patients	Parents
**Sex of patients**	n = 381	n = 615	**Vocational qualification**		n = 560
Female	50.4	48.0	Apprenticeship/training	-	51.8
Male	48.3	51.9	University degree	-	43.2
Diverse	1.3	0.2	No qualification	-	5.0
**Age of patients**	n = 365	n = 614	**School attendance**	n = 376	n = 576
1–5 years	-	5.0	High school	58.5	51.9
6–10 years	-	16.9	Secondary school	36.1	45.9
11–12 years	6.8	12.7	Elementary school	0.5	-
13 years	14.8	11.1	No longer in school/no graduation	4.8	2.3
14 years	20.5	16.1	**Career plans**	n = 369	
15 years	20.3	14.0	University	52.6	-
16 years	23.6	16.4	Apprenticeship	26.3	-
17 years	14.0	7.7	Other	15.7	-
**Living situation**	n = 377	n = 573	Voluntary/military service	5.4	-
Two-parents family	82.0	86.2	**Diagnosed IBD of patients**	n = 410	n = 613
Single mother or father	15.5	11.2	Crohn’s disease	52.4	51.2
In own home	0.3	-	Ulcerative colitis	42.2	40.8
Other	2.2	2.6	IBD unclassified	5.4	8.0
**Number of siblings**	n = 381	n = 570	**Diagnosed IBD of parents**		n = 619
				-	14.1
No siblings	16.3	19.5	**The current phase of the disease**	n = 403	n = 611
1–2 siblings	74.5	71.2	Inactive disease (remission)	75.7	68.4
3 or more siblings	9.2	9.3	Active disease (relapse/flare)	12.4	15.5
			Diagnostic phase	1.5	4.3
**Place of residence** (numbers of inhabitants)	n = 380	n = 570	Phase unknown	10.4	11.8
Rural community (<5 K)	31.8	29.3	**Course and pattern of progression**		n = 603
Small town (5 K–20 K)	27.1	27.9	Inactive disease (remission)	-	54.4
Medium-sized town (20 K–100 K)	19.7	20.9	Recurrent episodes of active and inactive disease	-	25.5
Large city (100 K–500 K)	10.0	10.9	Constantly active disease	-	17.4
Very large city (>500 K)	11.3	11.1	Disease flare	-	2.7
**Completed school qualification**		n = 567	**Duration of diagnosis**	n = 410	n = 616
High school	-	51.9	<1 year	13.4	18.2
Secondary school	-	31.6	1–2 years	27.6	28.1
Main school	-	4.6	3–4 years	23.9	22.7
**State**	n = 381	n = 571	5–6 years	13.2	12.2
North Germany (Bremen, Hamburg, Mecklenburg Western Pomerania, Lower Saxony, Schleswig-Holstein)	14.2	14.4	>6 years	22.0	18.8
			**Concomitant diseases**	n = 407	n = 594
			No concomitant disease	55.3	59.9
East Germany (Berlin, Brandenburg, Sachsen, Saxony-Anhalt, Thuringia)	21.0	17.8	Skin diseases	11.1	10.6
			Mental disorders	9.1	9.9
West Germany (Hesse, Northrhine-Westphalia, Rhineland Palatinate, Saarland)	37.5	36.8	Diseases of the joints	6.1	6.9
			Primary sclerosing cholangitis	5.2	4.9
South Germany (Baden-Württemberg, Bavaria)	27.3	31.2	Eye diseases, thrombosis, gall- and kidney stones	2.2	2.0
			I don’t know	13.3	-

**Table 2:** Utilization of options to cope with IBD from participating patients and patients whose parents responded to the CEDNA survey: Patients were asked which options they use (answer yes) and parents were asked how often in the past three months their child used the options to cope with the disease (answers never, rarely, regularly or often).

Responses in %							
Patients			Parents				
	n	Yes	n	Never	Rarely	Regularly	Often
Pursuing hobbies	380	84.7	582	8.4	13.4	46.0	32.1
Talking to your doctor about IBD	372	83.3	587	6.8	33.4	53.2	6.6
Meeting friends	376	83.2	583	7.5	19.9	43.4	29.2
Talking about IBD with family and/or friends	380	82.6	595	7.6	39.3	39.2	13.9
Doing sports and being physically active	370	74.3	595	7.6	39.3	39.2	13.9
Taking it easy on oneself	355	47.6	580	18.1	38.6	31.2	12.1
Paying special attention to the diet	354	38.7	586	17.4	33.8	33.1	15.7
Talking about IBD with other people affected	358	20.7	575	73.6	20.9	4.0	1.6
Performing relaxation exercises	356	19.1	567	65.8	27.5	4.8	1.9
Making use of psychological support	357	16.0	576	77.8	10.1	10.6	1.6
Participating in activities of a support group	359	0.8	579	97.4	1.7	0.7	0.2

**Table 3:** Level of knowledge and need for information about IBD, treatment and counseling services used by patients with IBD and their families, sources, and ways of information about IBD from the perspective of patients who participated in the CEDNA survey themselves and of patients whose parents participated in the CEDNA survey.

Responses in %													
Topics with Good Level of Knowledge (GL) and Topics with Need for Information (NI)									Ways of Information on IBD Preferred by Patients and Their Parents				
	Patients				Parents					Patients		Parents	
	n	GL	n	NI	n	GL	n	NI		n		n	
IBD in general	310	86.8	275	42.9	412	31.1	332	15.7	Websites	375	57.9	578	60.6
Nutrition	300	80.0	276	41.3	390	28.2	320	19.7	YouTube	375	54.7	576	22.9
Medical treatment options	300	74.0	284	44.0	376	18.1	332	20.8	Explanatory films	375	47.2	577	40.7
Vaccinations	303	68.0	275	33.8	398	27.1	321	11.5	Presentations	375	42.7	578	54.0
Medication side effects	302	65.2	284	38.0	393	19.8	324	17.6	Books for children	376	37.5	578	57.4
School and vocational training	303	64.0	281	47.3	380	18.2	315	20.3	One-day workshop	376	35.4	578	47.6
Prognosis	299	61.5	272	43.8	381	16.8	314	17.2	Apps with online information	374	35.3	576	27.3
Sexuality	297	52.9	266	27.8	380	12.9	302	4.2	Journals and expert books	376	33.2	578	48.8
Dealing with psychological stress	297	52.2	281	46.6	383	13.6	322	20.2	Information flyers	376	32.4	578	44.8
Causes	300	49.0	290	60.7	376	20.5	322	22.0	Forums for patients and/or parents	375	32.0	577	50.8
Psychotherapeutic measures	290	47.2	281	28.8	389	13.9	311	13.5	Apps as an online communication platform	375	28.3	578	18.3
Complications in the course	306	45.4	283	49.5	388	13.7	317	17.0	Blogs	375	25.3	577	10.7
Preventive health care	296	45.3	271	42.1	376	18.1	312	17.9	Chats	375	21.1	577	9.5
Concomitant diseases	302	40.1	284	51.4	386	11.1	320	18.1	Patient counseling services	375	17.3	577	41.1
Surgical treatment options	301	39.2	289	31.5	397	15.1	317	11.0	Seminar trips (overnight)	376	14.4	577	21.0
Patient organizations and Self-help groups	298	37.6	270	17.8	385	13.2	299	10.7	Regular newsletter	375	13.9	577	31.5
Travel abroad	298	34.6	282	45.0	379	11.9	306	15.0	Weekend seminars without overnight stay	376	10.9	578	15.4
Complementary medicine	294	29.6	280	36.1	370	13.0	311	23.5	Conferences	375	8.8	578	20.1
Social law issues	288	29.5	274	52.6	366	8.2	311	22.2	**Treatment and counseling services that have been used by patients and parents**				
Family Planning	-	-	-	-	377	14.3	299	12.7		**Patient**		**Parents**	
Transition	294	25.2	284	58.5	363	9.1	308	22.4		**n**		**n**	
**Sources of information and who should be giving information to patients and parents**									Nutritional counseling	382	49.2	575	46.1
Pediatric gastroenterologists	387	87.3			577	95.3			Psychological help/support	381	28.6	576	20.1
Specialized doctors for adults	386	56.2			576	40.6			Physiotherapy	381	15.7	576	8.0
Current researchers	387	40.8			577	43.2			Event to inform about the disease	381	10.5	576	22.2
Nutritionists	387	37.7			577	52.9			Family counseling	380	7.1	576	5.7
Other teenagers with IBD	387	33.9			577	38.5			Stress management offers	381	6.6	576	8.0
Psychologists	387	25.8			577	38.8			Ergotherapy	381	5.8	576	1.2
Affected families	387	23.0			577	39.7			Outpatient care service and home help	381	3.1	576	3.3
Nursing professionals	387	17.1			577	12.7			Advice on pension insurance	381	2.6	576	5.0
Sports professionals	387	14.7			577	14.2			Counseling through health or long-term care insurance	381	2.4	576	4.2
Patient associations	387	12.7			577	22.2							
Support groups	387	7.2			577	18.4			Genetic counseling	381	2.1	576	6.1
Social workers	387	5.7			577	9.0			Transition programs	381	1.3	576	0.9
Lawyers	387	3.4			577	6.4			Sexual counseling	381	0.8	576	0.3
Health insurance companies	387	3.1			577	14.0			Family Planning Counseling	-	-	576	0.5
Music educators	387	2.1			577	6.8			Marriage Counseling	-	-	576	0.5
Consumer protection centers	387	1.8			577	8.1			Do not use or benefit from any treatment or counseling service	379	31.4	575	35.0

**Table 4:** Responses on trustworthiness and utilization of sources of information on IBD by patients who participated in the CEDNA survey (responses: Yes, the source is trustworthy and I inform myself through these sources) and of the patients whose parents responded to the CEDNA survey (responses: Yes, the source is trustworthy, very/mostly/only a little/not at all trustworthy and I inform myself through these sources).

Information Sources	Patients				Parents						
	n	Yes, Trustworthy	n	Source: I Inform Myself through These Sources	n	Very Trustworthy	Mostly Trustworthy	Only a Little Trustworthy	Not at All Trustworthy	n	Source: I Inform Myself through These Sources
Responses in %											
Doctors	310	98.7	276	90.2	295	58.1	37.0	4.1	0.7	351	96.0
Professional journals, specialist books	273	79.9	268	42.5	366	22.2	43.0	20.2	14.6	300	64.0
Medical societies	268	78.0	261	17.2	408	22.3	37.0	23.3	17.3	278	31.3
Nutritionists, nutrition consultants	275	74.5	267	43.1	373	16.4	39.7	26.9	17.0	292	46.2
Families, friends, acquaintances	281	70.8	266	62.4	365	15.7	28.3	39.4	16.6	288	48.3
Other patients	271	67.2	266	34.2	399	13.9	33.0	32.0	21.0	284	36.6
Pharmacist	283	65.0	267	21.0	358	12.3	33.7	36.3	17.7	308	32.1
Patient Associations	255	62.4	267	22.1	391	34.4	30.3	16.1	19.2	291	56.0
Psychologists	266	62.0	265	23.0	392	13.6	36.7	25.6	24.1	285	32.6
Support groups/Self-help groups	244	47.1	265	5.7	429	11.8	33.0	19.4	35.8	282	14.5
Health insurance companies	253	45.1	259	7.7	386	5.0	29.5	40.1	25.5	285	20.7
Internet in general	267	40.8	275	56.7	346	8.8	25.3	39.2	11.6	303	72.9
Alternative practitioners	258	40.3	258	21.7	378	5.8	23.3	36.4	34.5	288	28.5
Pharmaceutical industry	251	32.3	257	5.8	406	2.6	19.2	47.0	31.1	284	15.1
Special internet forums, chatrooms	256	31.6	267	19.5	396	4.8	40.3	38.8	31.1	289	31.8
Transition programs	224	31.3	257	1.6	492	6.9	18.5	19.9	54.6	261	6.1
Television, consumer programs	257	28.0	265	20.8	378	3.6	21.2	44.5	30.6	289	36.0
Politics	252	14.7	259	3.5	412	0.7	2.7	28.7	67.9	280	3.9

**Table 5:** Assessing when information on specific IBD topics is or is not needed by **parents** of children with IBD who participated in the CEDNA survey.

Information Needed at What Time	n	At Diagnosis	In the First Year	In the Further Course	No Need for Information
Topics IBD	Responses in %				
IBD general	552	67.6	10.5	17.0	4.9
Causes	535	63.2	16.3	15.9	4.7
Nutrition	538	49.8	22.1	21.9	6.1
Medical treatment options	555	47.7	16.8	32.1	3.4
Medication side effects	542	45.2	21.2	28.4	5.2
Prognosis	536	37.3	20.5	36.6	5.6
Dealing with psychological stress/stress management	532	33.1	26.1	32.7	8.1
Psychotherapeutic accompanying measures	526	31.6	22.6	33.8	12.0
Concomitant diseases	540	29.1	25.4	41.7	3.9
Complementary medicine	511	28.6	23.5	34.8	13.1
Vaccinations	523	28.1	27.9	33.7	10.3
Patient organizations and self-help groups	489	25.8	24.1	31.7	18.4
Disease documentation	492	24.4	27.2	35.4	13.0
School and vocational training	528	24.2	28.2	40.3	7.2
Measures for preventive health care	509	21.4	23.6	45.0	10.0
Complications in the progressive course of the disease	532	19.7	19.4	56.0	4.9
Surgical treatment options	489	14.9	11.8	58.8	14.5
Social law issues	509	11.0	16.7	63.5	8.8
Travel abroad	475	10.1	20.2	49.7	20.0
Family planning	475	3.6	2.1	69.7	24.6
Transition to adult medicine	525	3.6	4.4	84.2	7.8
Sexuality	484	3.3	3.7	70.5	22.5

## Data Availability

Restrictions apply to the availability of these data. The data were obtained from the quality improvement project CED-KQN (Big Data eHealth—Improving the Care of Children and Adolescents with IBD) which was funded by the Federal Joint Innovation Committee G-BA and will be available on the G-BA’s URL https://innovationsfonds.g-ba.de/projekte/versorgungsforschung/ced-kqn-big-data-ehealth-verbesserung-der-versorgung-von-kindern-und-jugendlichen-mit-chronisch-entzuendlichen-darmerkrankungen.171 (accessed on 31 October 2023) from 1 January 2024.
